# Synthesis and 3D Printing of Conducting Alginate–Polypyrrole Ionomers

**DOI:** 10.3390/gels6020013

**Published:** 2020-04-18

**Authors:** Cassandra J. Wright, Binbin Zhang Molino, Johnson H. Y. Chung, Jonathan T. Pannell, Melissa Kuester, Paul J. Molino, Timothy W. Hanks

**Affiliations:** 1 Department of Chemistry, Furman University, Greenville, SC 29613, USA; cassandra.wright@gcu.edu (C.J.W.); pannell@musc.com (J.T.P.); mkuester01@gmail.com (M.K.); 2 ARC Centre of Excellence for Electromaterials Science, Intelligent Polymer Research Institute, AIIM Faculty, University of Wollongong, Wollongong, NSW 2522, Australia; bz107@uowmail.edu.au (B.Z.M.); johnsonc@uow.edu.au (J.H.Y.C.); pmolino@uow.edu.au (P.J.M.)

**Keywords:** conducting polymer, biopolymer, oxidative polymerization, extrusion printing, tissue scaffold

## Abstract

Hydrogels composed of calcium cross-linked alginate are under investigation as bioinks for tissue engineering scaffolds due to their variable viscoelasticity, biocompatibility, and erodibility. Here, pyrrole was oxidatively polymerized in the presence of sodium alginate solutions to form ionomeric composites of various compositions. The IR spectroscopy shows that mild base is required to prevent the oxidant from attacking the alginate during the polymerization reaction. The resulting composites were isolated as dried thin films or cross-linked hydrogels and aerogels. The products were characterized by elemental analysis to determine polypyrrole incorporation, electrical conductivity measurements, and by SEM to determine changes in morphology or large-scale phase separation. Polypyrrole incorporation of up to twice the alginate (monomer versus monomer) provided materials amenable to 3D extrusion printing. The PC12 neuronal cells adhered and proliferated on the composites, demonstrating their biocompatibility and potential for tissue engineering applications.

## 1. Introduction

Intrinsically conducting polymers (ICPs) [1], including polypyrrole (PPy), polyaniline (PANi), and poly(3,4-ethylenedioxythiophene) (PEDOT), have been in the materials science toolbox for decades and are of interest in a variety of fields including organic electronics and optoelectronics [2]. Intrinsically conducting polymers also offer a suite of unique properties that are particularly valuable in the emerging area of bioelectronics or “bionics”, involving the communication between biological systems and electronic devices [3,4]. They are both ionically and electronically conducting, allowing for electrochemical redox processes to drive ions into and out of a structure. They can be cheaply fabricated by chemical or electrochemical processes into films, nanostructures, and bulk materials. Their molecular structures can be modified either prior to or after polymerization, and they often show good biocompatibility and low toxicity. Thus, ICPs are under active investigation for diverse applications in biomedical engineering [5] including biosensing [6], cellular interfacing [7], controlled drug release [8], and tissue engineering [9,10,11].

The most widely used ICPs are polycations in their conducting forms. The simple parent polymers are insoluble and intractable when prepared with standard inorganic counterions, necessitating the development of other approaches to improve processability, biocompatibility, and physical properties for particular applications. There are two primary strategies for this: derivatization of the ICP backbone or changing the anionic counterion (usually called the “dopant”). The ICP derivatization usually involves the addition of groups that improve solubility or add new functionality to the monomers. While useful, the synthesis of such species introduces extra complexity and cost and may negatively impact non-target physical and electronic properties [12]. The search for alternative dopants has also proven to be a fertile area of investigation. Early work focused on dopants, such as dodecylbenzene sulfonic acid, camphor sulfonic acid, and others, which helped to improve solubility/dispersibility in organic solvents and, in some cases, also enhanced conductivity. As with ICP derivatization, each dopant broadly impacts on the properties of the resulting material. The electrical conductivity, for example, is generally decreased as the mass of the non-conductive components increases. In addition to small molecule dopants, ICP composites have been reported based on anionic polymers, including polyacrylates [13], poly(sulfonic acids) [14], and various biopolymers [15,16].

The role of polyanions in ICP ionomeric composites varies widely and is not always entirely clear. Often, a small molar ratio of a polyanion serves as templates to control the morphology of the resulting material, while the bulk of the charge balancing is due to the fact of other anions. In other cases, biopolymers in the form of hydrogels simply form a matrix or template, within which conventional ICP polymers can be grown or deposited [17,18,19]. For example, recent reports have described the formation of cross-linked alginate hydrogels in which PPy or PANI have been electrochemically deposited. As in most composites that have been reported, the biopolymer does not act as the dopant but serves as a structural component. Recent reviews describe several important uses of ICP/hydrogel composites including cellular scaffolds for tissue engineering, interfaces between electrodes and neural tissue, biosensors, and others [9]. These “wet” ICP structures are quite different from the “dry” films and powders of the early work in the ICP field, and they offer distinct advantages when interfacing with biological systems [20].

Sodium alginate is a carbohydrate obtained from brown algae and widely used in the food industry as a stabilizer, emulsifier, and gelling agent [21]. It is also useful in applications ranging from drug delivery to tissue engineering [22] due to the fact of its good biocompatibility and non-immunogenicity. The viscoelastic properties of alginate can be varied dramatically by cross-linking with multiply charged metal ions, such as Ca^2+^, chemical cross-linking, or by blending with polycations. It is not only highly biocompatible but is bio-erodible, since the gelation is reversible, yielding soluble dispersions when the polyvalent cross-linker is exchanged for monovalent ions. Alginate can easily be fabricated into fibers, foams, nanoparticles, films and extruded [23], spun [24] or printed as free-standing 3D structures [25]. One limitation of alginate as a bioink, however, is that it lacks cell adhesive moieties and requires chemical modification or additives for improved cellular interactions and is not well suited for applications requiring electrical stimulation of cells [26,27]. 

The PPy-based composites are among the most widely studied ICP materials for biomedical applications. Polypyrrole is highly biocompatible and has been shown to support the adhesion and growth of many cell lines. In addition, we have shown that the polymer can undergo efficient reactions with thiols, providing an interesting post-polymerization method for surface modification to further control cellular adhesion [16]. As the pyrrole monomer is highly water soluble, formation of hydrophilic composites is facile. Previous preparations of ICP/alginate composites have focused primarily on the use of the biopolymer as a template leading to the formation of nanofibers or particles [17,19]. The preparation of a highly polypyrrole-rich polypyrrole/alginate (PPy–ALG) composite for use as an ink for 3D printing has not been explored. This approach presents significant advantages, including inherent electroactivity through the bulk polymer (as opposed to forming through incorporation of ICP nanoparticle networks within a base polymer), and the ability to tailor and modulate polymer composite physicochemical, mechanical, and electrical properties through varying the polymerization conditions. However, as shown in this report, care must also be taken to prevent the degradation of the alginate polymer during the PPy–ALG polymerization, highlighting the need to control the polymerization conditions to produce a biocomposite that presents the desired properties of both the PPy and the alginate dopant species. Here, we demonstrate the preparation of PPy–ALG composites that possess both the electronic conductivity and cell adhesive properties of PPy and the flexible processability of alginate. The PPy/alginate composites are readily dispersed in aqueous solutions and form hydrogels by cross-linking with Ca^2+^. We further demonstrate the suitability of the composites as bioinks by printing the material into free-standing scaffolds. Combined with our previously reported work on PPy surface modification, this provides a potential route to spatially controlled cell growth platforms [16,28,29,30].

## 2. Results and Discussion

### 2.1. Optimization of PPy–Alg Composite Synthesis

Polypyrrole is generally formed by the chemical or electrochemical oxidation of pyrrole. The most widely used chemical oxidants are the ferric salts, though the reaction works with other oxidizers, such as persulfates, as well [31]. Persulfate salts are inexpensive, but the reaction rate of pyrrole oxidation is slow. Therefore, it is generally activated by the addition of a small amount of a secondary oxidant, such as ferric chloride, to form the kinetically more aggressive sulfate radical. However, transition metal salts are not only difficult to remove from alginates, but they can display toxicity and are therefore undesirable in biomedical applications. In addition, the physical properties of alginic acid salts are highly dependent on the identity of the cationic counterion. Alginates are particularly sensitive to the presence of multivalent cations which act to cross-link polymer chains. In anticipation that undesired metal ions would be difficult to remove from a pyrrole–alginate composite, sodium persulfate (NaPS) was used as the sole oxidant in these studies. 

In initial experiments, light yellow solutions of sodium alginate were prepared by adding distilled water and stirring until completely dissolved. Upon addition of pyrrole and NaPS, the solution gradually darkened until it was jet black. No precipitate was formed, but it was observed that the solutions became markedly less viscous as the reaction proceeded. Additionally, calcium cross-linking proved to be far less effective than with pure alginate. 

**Figure 1:**
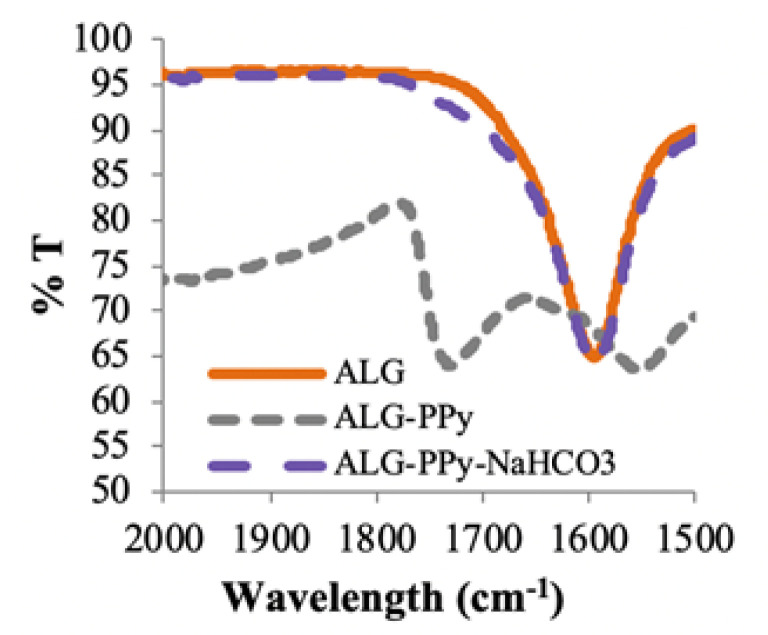
FTIR data of the carbonyl region showing alginate, PPy–Alg, and PPy–Alg reacted in the presence of NaHCO_3_.

Upon drying, the composite produced highly brittle, amorphous films. The ATR-IR spectroscopy (Figure 1) of these films showed a carbonyl peak at 1725 cm^−1^ that is not present in either PPy grown by conventional methods or pure alginate, indicating that the production of PPy was causing oxidization of the polysaccharide. However, treatment of pure alginate with the NaPs oxidant under the reaction conditions did not result in the formation of the carbonyl. These results suggested that the pyrrole polymerization reaction itself caused the decomposition of the alginate film.

Methods to encourage the partial decomposition of alginate have been developed by several workers, primarily as a way to increase rates of biodegradation. For example, heating of alginate in the presence of silver ions, hydrogen peroxide [32] or potassium persulfate decreases polymer chain length through hydrolysis [33], as does exposure to gamma irradiation [34]. In this system, conducting the pyrrole polymerization in the presence of EDTA to scavenge possible incipient metal ions had no effect on the reaction. Running the reaction on an ice bath slowed the overall rate of polymerization but did not prevent the formation of a carbonyl or the reduction in solution viscosity.

**Scheme 1:**
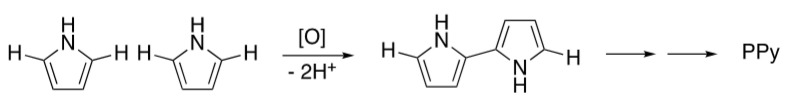
Mechanism for the oxidative polymerization of pyrrole.

Persulfates have been shown to be activated by either low pH or high pH, particularly at elevated temperatures [35]. The activation results in the formation of other oxidizing species that have lower oxidizing power but can have faster reaction rates. Since the oxidative polymerization of pyrrole (Scheme 1) results in the production of protons, we found that the pH of the reacting solution dropped throughout the course of the reaction. Simply preventing the pH from dropping below 4 prevented alginate hydrolysis and resulted in stable solution viscosities and materials that could be efficiently cross-linked with calcium ions. Several buffers and mild bases were shown to prevent oxidative hydrolysis with varying degrees of effectiveness (data not shown). Ultimately, NaHCO_3_ was found to be highly effective, easy to use, and easy to remove from the product by dialysis.

**Figure 2:**
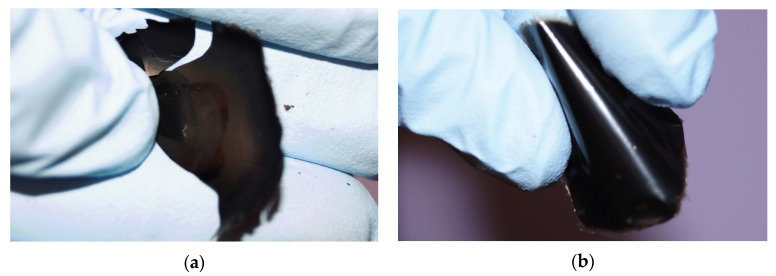
Cross-linked PPy–Alg films prepared from composite A (**a**) without and (**b**) with NaCO_3_.

The effect of pH control on the composite material can be clearly seen from cast films prepared from solutions with an initial 1:1 ratio of pyrrole to alginate either without or with NaHCO_3_. As can be seen in Figure 2, the films prepared without the base were quite brittle, where the films prepared at a pH above 4 were easily bent without damage.

### 2.2. Characterization of Composite Materials

**Figure 3:**
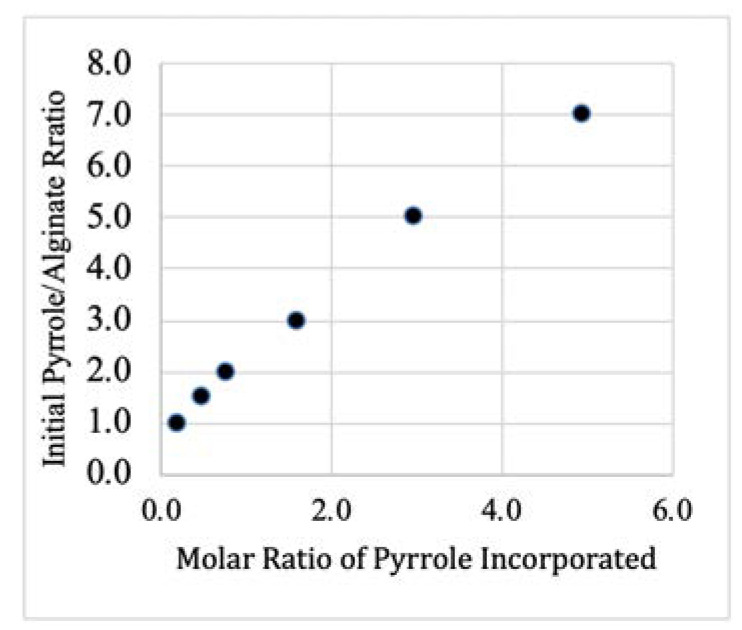
Incorporation of pyrrole into PPy–Alg composites prepared from solutions with different molar rations of pyrrole to alginate. Data based on combustion analysis. Compositions from lowest to highest PPy content are referred to as composites B–F, respectively.

Six PPy–Alg composite formulations (A–F) were prepared. The initial molar ratios of pyrrole to alginate monomer prior to polymerization were set at A = 1:1, B = 1.5:1, C = 2:1, D = 3:1, E = 5:1, and F = 7:1. Combustion elemental analysis was used to determine the actual ratio of PPy to alginate in the resulting composites. The PPy content was calculated based on the percentages of nitrogen and carbon in the composites. Since the only nitrogen in the sample came from the pyrrole, the carbon content due to the pyrrole could be calculated based upon the percent nitrogen to the total mass. The remaining carbon was presumed to be from the alginate allowing for the calculation of the PPy/Alg ratio. As expected, the results from this analysis (Figure 3) showed that the amount of pyrrole incorporated into the composite increased as the initial concentration of the monomer increased. Interestingly, at the lowest pyrrole concentration (composite A), only approximately 20% of the pyrrole was incorporated into the composite, while composite F was found to retain more than 70% of the initial pyrrole. During the dialysis purification step, we observed that significant amounts of a brown material was extruded from the dialysis tubing. This material was almost certainly pyrrole oligomers. It is likely that at low pyrrole concentrations the coupling reaction was inefficient, resulting in short highly soluble oligomers that were only loosely adhered to the alginate. At higher concentrations, the chains were able to grow longer to the point where they aggregate and more PPy was retained in the final product.

Lyophilization of gels from composites A–D resulted in elastic, highly porous sponges. Even at low PPy concentrations, the dried material was markedly less brittle than the pure sodium alginate treated under the same conditions. With compositions E and F, lyophilization resulted only in dried films. The SEM images (Supplementary Materials Figure S1) for composite C show a morphology similar to that of sodium alginate, with rough, sheet-like structures supported with fibers. Sodium alginate dissolves slowly in water and the 2% *w*/*v* solutions used in this study are quite viscous. As the PPy concentration increased, the dried sponges were found to dissolve much more quickly and gave progressively less viscous solutions. 

**Figure 4:**
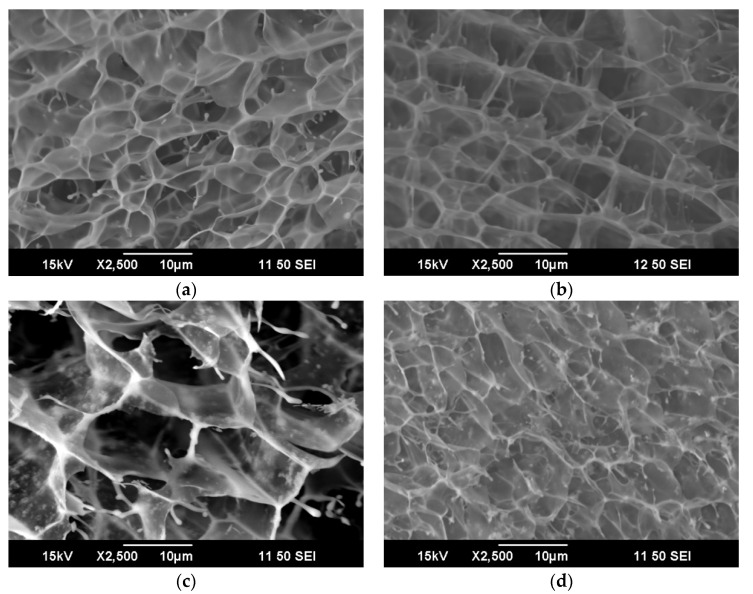
SEM images showing the pore structure of each of the substrates: (**a**) alginate, (**b**) composite A, (**c**) composite B, (**d**) composite C. Scale bar 10 µm.

Treatment of alginate solutions with divalent cations, particularly Ca^2+^ results in cross-linking of the polysaccharide chains and the formation of gels. So too, addition of calcium ions resulted in PPy–Alg gel formation. Interestingly, the polycationic pyrrole chains do not have the same effect, likely because the tightly bound “egg-box” structure is not formed. The rate and homogeneity of the cross-linking is dependent on the calcium source. As has been previously described, cross-linking of calcium carbonate is particularly effective when the rate is moderated by the addition of D-glucono-δ-lactone (GDL) [36]. At low PPy concentrations (composites A–C) comparisons of SEM micrographs of Ca^2+^ cross-linked alginate and PPy–Alg reveal a very similar open pore structure (Figure 4) with pore diameters of 5–10 µm (probably due to the size of the ice crystals formed rather than an inherent property of the hydrogel). There was no evidence of phase separation or the formation of nanoparticles typically found with PPy prepared with conventional dopants. As the PPy concentration increased, the gels progressively weakened. The SEM image of composite C (Figure 4d) already showed loss of pore definition, and the gel from composite D showed only an amorphous material. Composite E was able to form a very weak gel, but composite F simply formed a flocculent precipitate. This behavior was not unexpected, since PPy is a polycation and increasing its overall contribution to the composite necessarily results in fewer available sites for the Ca^2+^ ions to effect cross-linking. In its conducting oxidation state, PPy has approximately one positive charge for every three rings, so composite E would be expected to be roughly electrically neutral without additional ions.

**Figure 5:**
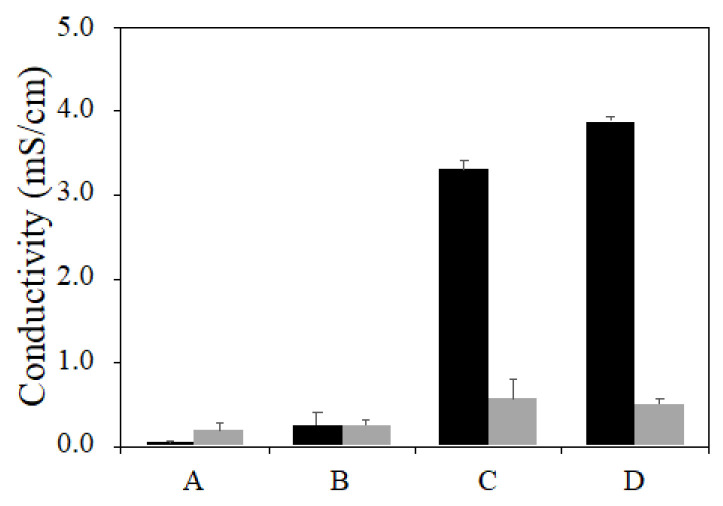
Conductivity of non-cross-linked (black) and cross-linked (grey) PPy–Alg thin films of composition A, B, C, and D measured via the four-point probe method.

While dried alginate is an insulator, the incorporation of PPy permits the formation of electrically conducting films. Composites of composition A–F were prepared and purified as usual. Solutions of the PPy–Alg sodium salts as well as gels cross-linked with calcium were cast and dried at atmospheric pressure to form thin films. The electrical conductivity was measured by the four-point probe method (Figure 5). Not surprisingly, increasing the PPy content of the composite increased the observed conductivity, though the cross-linked films displayed only modest differences. With the films prepared from the non-cross-linked materials, the conductivity increased by an order of magnitude going from composite B to C, suggesting a key percolation threshold had been reached. The conductivity differences between the cross-linked and un-cross-linked films is a function of the change in the balance of intrinsic ion pairs (polymer–polymer) versus extrinsic pairs [37]. We propose that the Ca^2+^ ions cause phase separation of the polyelectrolytes and reduce the effectiveness of electron chain-transfer. 

The conductivity of films with high concentrations of PPy (E and F) were too brittle to analyze. They were observed to fragment during the drying stage or during attempts to apply the four-point probe to their surfaces. 

Cross-linked alginate gels conduct electricity through the movement of mobile ions within the gel. In order to examine the influence of PPy incorporation into the composite gels, the electrical impedance behavior of the hydrogels was determined as previously described [38], where the resistive component of the gel impedance magnitude is determined using equivalent circuit modelling. Table 1 shows that the conductivity of pure cross-linked alginate as compared to gels of composition A, B, and C. Clearly, the ionic conductivity dominated the electrical behavior of the gel, with only minor changes across the samples. At higher concentrations of PPy, the number of ions available as charge carriers decreased, resulting in lower overall conductivity despite the likely increase in the contribution of electronic conductivity. Gels made from compositions D, E, and F were too fragile to measure.

### 2.3. Extrusion Printing

**Figure 6:**
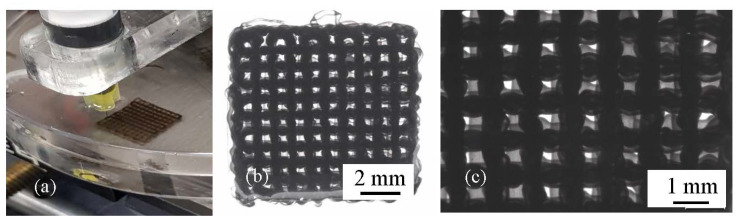
Structure of printed scaffolds using composite B: (**a**) extrusion printing of ink; (**b**) low magnification (10×); (**c**) high magnification (30×).

The PPy–Alg inks were assessed for their printability using a 3D extrusion printer. Table 2 shows the scaffold dimensions that were observed as compared to those in the original design. The theoretical filament width is based on the extrusion needle, while the filament spacing is a function of the printer precision and the motion of the deposited filaments. The smaller pore size and wider filament width compared to the scaffold design shows that under the extruding conditions this ink spreads within the printing bath solution. The spacing of the filaments, however, was well controlled. Attempts to limit ink spreading by early exposure to increased Ca^2+^ resulted in the filaments adhering to the deposition needle, dramatically decreasing printing fidelity. The spreading of individual filaments can be accounted for during the pre-printing step by adjusting the strand spacing and layer thickness to result in a scaffold with overall dimension similar to the intended size of 10 mm × 10 mm (Figure 6).

Preliminary cell culture experiments demonstrate that adhesion of PC12 cells to the PPy–Alg is comparable to, or slightly better than, alginate alone with no evidence of cytotoxicity of the PPy–Alg hydrogel (Figure S2). An increase in calcium concentration was needed for cell culture above the standard protocol to ensure the calcium would remain within the scaffolds and not be replaced by sodium, a process that would eliminate the cross-linking and structure of the gel. These results indicate that PPy–Alg presents good cytocompatibility that is comparable to alginate alone, presenting this novel, electroactive material suitable for biomaterial and tissue engineering applications.

## 3. Conclusions

Ionomeric composites of PPy and alginate have considerable potential for applications requiring cellular scaffolding. Here, PPy/Alg ratios ranging from approximately 0.2 to 5 were prepared and characterized showing the scope as well as some limitations to this approach. At lower PPy concentrations, the materials behaved much like pure alginate, except that the dried films displayed enhanced electrical conductivity. At ratios of PPy above 2:1, the materials become increasingly mechanically fragile and unusable for most potential applications. Composites with equivalent amounts of PPy and alginate are suitable for 3D extrusion printing and displayed no increased toxicity to PC12 cells; however, no increase in electrical conductivity was observed in the swollen gels. The Alg component of these ionomers provides the structural properties useful for scaffold formation, while the PPy opens up the potential for spatially controlled surface modification to rationally control cellular adhesion and growth.

## 4. Materials and Methods 

### 4.1. General

Alginic acid sodium salt, pyrrole, pyridine, sodium carbonate, d-glucono-δ-lactone (GDL), and sodium persulfate (NaPS) were purchased from Sigma–Aldrich and used as received. Pyrrole was distilled and stored at −20 °C in an airtight container until use. Water for synthetic reactions and dialysis was deionized and purified on a Milli-Q system (EMD Millipore, Billerica, MA, USA) to Type 1 grade prior to use. Ethylenediaminetetraacetic acid (EDTA) and sodium bicarbonate were purchased from Fisher Scientific (Fair Lawn, NJ, USA) and used as received. Membra-Cel MD77 regenerated cellulose dialysis membrane (MWCO = 12–14 kDa), purchased from SERVA (Heidelberg, Germany), was used for sample purification. The pH of product solutions was measured using an Orion VersaStar pH meter (ThermoScientific, Waltham, MA, USA). Attenuated total reflectance infrared spectroscopy (ATR-IR) data were collected on cast films using the ATR method on a Perkin Elmer Frontier spectrometer. Conductivity measurements on the films were made using a Jandel four-point probe and RM3 resistivity meter (Bedford, UK).

### 4.2. Synthesis of 2% Alginate Control

Alginate solutions (2% *w*/*v*) were prepared by adding alginic acid sodium salt (1.00 g) to water (50 mL) and stirring at room temperature for 24 h, allowing alginic acid to completely dissolve. The solutions were then processed by dialysis against water for 48 h. Excess water was removed under vacuum to return the solutions to 2% *w*/*v*, and samples were stored at 4 °C until needed. Alternately, samples were lyophilized and stored until needed, at which time they were then rehydrated to 2% *w*/*v* concentration. The pH and viscosity of all solutions were recorded. Cast films of the composites were generated by dehydrating 3 mL of each sample in a vacuum oven at 35 ± 5 °C for 48 h. 

### 4.3. PPy/Alginate Composites (PPy–Alg), General Method

Alginate solutions (2% *w*/*v*) were prepared by adding alginic acid sodium salt (1.00 g) to water (50 mL) and stirring for 2 h, allowing alginic acid to completely dissolve. Pyrrole (1.0–7.0 molar equivalents versus saccharide monomer, as indicated in specific experiments), NaPS (2.5 molar equivalents versus pyrrole), and NaHCO_3_ (4.0 molar equivalents versus pyrrole) were added and the solution stirred either on an ice bath or at room temperature for 4 h, allowing in situ polymerization of the pyrrole in alginate (PPy–Alg). The resulting black solutions were then purified by dialysis against water for 48 h. Excess water was removed under vacuum to return the solutions to 2% *w*/*v* PPy–Alg, and samples were stored at 4 °C until needed. Alternately, samples were lyophilized for storage and then rehydrated to 2% *w*/*v* concentration as needed.

### 4.4. Calcium Cross-Linking

Cross-linked hydrogels were prepared with a slow cross-linking procedure previously reported [36]. Briefly, 205 mg of calcium carbonate (2.05 mmol) was stirred added into 40 mL of 2.5% *w*/*v* alginate solutions. A second solution consisting of GDL (728 mg, 4.09 mmol) dissolved in 10 mL of water was briefly vortexed. The two solutions were vigorously mixed yielding a 2% *w*/*v* alginate solution. This was immediately poured into molds and allowed to gel for at least 24 h at room temperature or used for printing as described below.

### 4.5. Elemental Analysis

Elemental analysis was performed by the Macquarie University Chemical Analysis Facility, Sydney, to assess the resultant ratios of carbon, nitrogen, hydrogen, and sulfur. A small amount of 0.1 M sulfuric acid was added to 2 mL samples of each concentration of PPy–Alg to remove any residual sodium bicarbonate still present in the system followed by 24 h of dialysis. Samples were then lyophilized.

### 4.6. SEM Analysis

A JEOL JSM-6490LA analytical scanning electron microscope (SEM) was utilized to image the hydrogels using an acceleration voltage of 15 kV and a cold stage technique. Data were collected on lyophilized un-cross-linked materials, snap-frozen cross-linked gels, and lyophilized cross-linked gels. The lyophilized gels were coated with a thin conducting layer of gold and immobilized on a stainless-steel stage. The frozen hydrogels were fractured to obtain undamaged cross-sections for imaging. 

### 4.7. Electrical Measurements

Electrical conductivity was assessed using two methods: four-point probe and impedance. The four-point probe (Jandel RM3, Leighton Buzzard, UK) method was used to assess the conductivity of dried films. Electrical impedance behavior of the hydrogels was determined using a custom apparatus, previously described [38], that utilizes frequencies ranging from 1 to 100 kHz and sample lengths ranging from 0.5 to 2.5 cm in 0.5 cm increments. Briefly, the conductivity was determined using a previously established method [33], where the resistive component of the gel impedance magnitude is determined using equivalent circuit modelling. Alginate and PPy–Alg solutions were poured into acrylic sample holders containing two pieces of reticulated vitreous carbon (RVC, ERG Aerospace, 20 pores per inch) and allowed to cross-link using the method described above. An alternating current signal (0.8 V peak voltage) was applied using a waveform generator (Keysight Technologies U2761A, Santa Rosa, CA, USA) across a known resistor (10 kΩ) in series with the hydrogel sample. The current was determined using the difference in electrical potential across the known resistor using an oscilloscope (Agilent U2701A). The impedance across the gel sample was calculated using the applied current and the measured current. 

### 4.8. Fabrication of Scaffolds

The PPy–Alg ink was extruded onto a glass slide immersed in a 15% *v*/*v* EtOH/distilled water solution using a KIMM (Korea Institute of Machinery & Materials, (Daejeon, Korea) 3D printing system. The system was equipped with a three-axis positioning platform and the printing pattern designed using the M4T software. A custom laser cut attachment for syringe deposition was fabricated and connected to the moving head of the printer. The ink solution was loaded into a 3 cc disposable syringe (Nordson EFD, East Providence, RI, USA) and fitted with a 100 µm diameter nozzle. Ten layers of the ink solution were extruded as a lattice at a feed rate of 140 mm/min, layer thickness of 0.1 mm, corner acceleration of 90%, and strand spacing of 1 mm, to a final size of 10 mm × 10 mm. Pneumatic air pressure was applied for extrusion and optimized at 5 kPa. Immediately after printing, 5% *w*/*v* CaCl_2_ was added in dropwise to ionically cross-link the scaffold. The macroscopic structure of extruded scaffolds was imaged using Leica M205A optical microscope (Leica Microsystems, Wetzlar, Germany).

### 4.9. Cellular Adhesion and Growth

The PC12 cells were cultured in an incubator (37 Pyrrole was distilled and stored aC, 5% CO_2_) in proliferation media containing DMEM supplemented with 10% horse serum, 5% fetal bovine serum, and 1% penicillin–streptomycin. Media had amended calcium content to 5 mM to increase the availability of calcium ions for maintaining the cross-linking. 

Hydrogels were poured and cross-linked directly into 12 well tissue culture plates and allowed to cross-link for two days. After cross-linking was complete, hydrogels were sterilized by soaking in 70% EtOH, followed by evaporation of the remaining EtOH under sterile conditions in a biosafety cabinet. Following sterilization, hydrogels were soaked in calcium-amended DMEM for 2 h prior to cell seeding. Hydrogels were seeded at a density of 3000 cells/cm^2^ and incubated for 48 h. 

Live-dead fluorescent staining was used for analysis. Cells were fixed in 3.7% paraformaldehyde at room temperature for 10 min. Cells were then stained with calcein (1:200 dilution) and propidium iodide (1:1000 dilution). Imaging was performed on an AxioImager (Zeiss, Oberkochen, Germany) fluorescence microscope with Axiovision SE64 software. 

## Figures and Tables

**Table 1:** Conductivity of cross-linked alginate and composite gels.

Composite	Conductivity (mS/cm)	Error (mS/cm)
Alginate	5.25	0.45
A	6.33	0.27
B	4.41	0.18
C	4.07	0.20

**Table 2:** Dimension of printed structures from composite B.

Dimension	Actual	Theoretical
Pore width (mm)	0.41 ± 0.04	1
Filament width (mm)	0.58 ± 0.06	0.1
Filament spacing (mm)	1.04 ± 0.03	1

## Supplementary Materials

The following are available online at https://www.mdpi.com/2310-2861/6/2/13/s1, Figure S1: SEM micrograph of cross-linked cryogel of composite A; Figure S2: Representative live (green)/dead (red) fluorescence images of PC12 neuronal cells on alginate.
