# Supplementary material for: Synthesis and 3D Printing of Conducting Alginate–Polypyrrole Ionomers
Source: Gels. 2020 Apr 18;6(2):13. doi: 10.3390/gels6020013 (PMC7344549; doi:10.3390/gels6020013)
Supplement: Supplementary file 1 [file gels-06-00013-s001.pdf]

Supplemental Materials

Synthesis and 3D Printing of Conducting Alginate-Polypyrrole Ionomers

*Cassandra J. Wright, Binbin Zhang Molino, Johnson H. Y. Chung, Trent Pannell, Melissa Kuester, Paul J. Molino, Timothy W. Hanks**

Supplemental Figures:

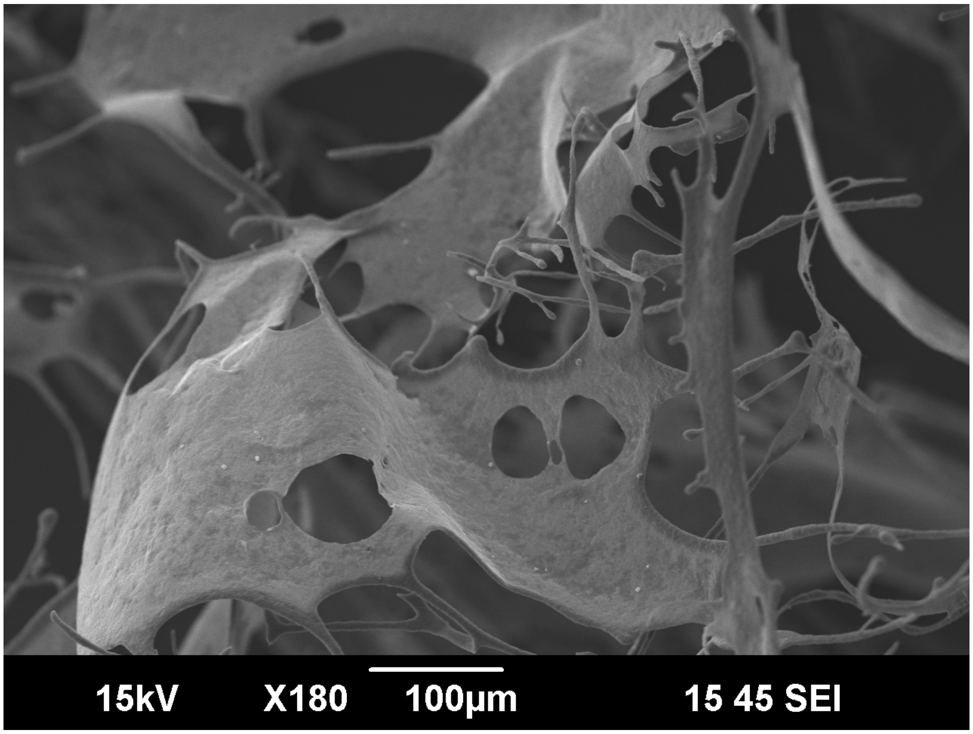

Figure S1: SEM micrograph of cross-linked cryogel of composite **A**.

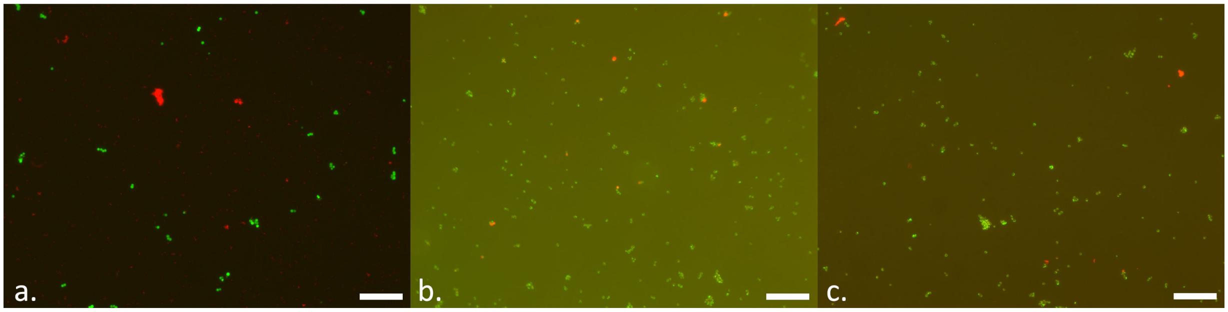

Figure S2: Representative Live (green)/Dead (red) fluorescence images of PC12 neuronal cells on alginate (a) and PPy-Alg composites A and B (b,c) hydrogels. Scale bars represent 100 µm.
